# Niclosamide ethanolamine ameliorates diabetes-related muscle wasting by inhibiting autophagy

**DOI:** 10.1186/s13395-021-00272-7

**Published:** 2021-06-09

**Authors:** Yuchun Cai, Hongyue Zhan, Wenci Weng, Yao Wang, Pengxun Han, Xuewen Yu, Mumin Shao, Huili Sun

**Affiliations:** 1 Department of Nephrology, Shenzhen Traditional Chinese Medicine Hospital, The Fourth Clinical Medical College of Guangzhou University of Chinese Medicine, 1 Fuhua Road, Futian District, Shenzhen, 518033 Guangdong China; 2 Department of Critical Care Medicine, Shantou Hospital of Traditional Chinese Medicine, Shantou, China; 3 Department of Pathology, Shenzhen Traditional Chinese Medicine Hospital, The Fourth Clinical Medical College of Guangzhou University of Chinese Medicine, 1 Fuhua Road, Futian District, Shenzhen, 518033 Guangdong China

**Keywords:** Niclosamide ethanolamine salt, Diabetes-related muscle wasting, Autophagy

## Abstract

**Background:**

Diabetes-related muscle wasting is one of the devastating complications of diabetes, which is associated with muscle autophagy due to insulin-mediated glucose starvation. However, treatment for diabetes-related muscle wasting is limited. Our previous study already found that niclosamide ethanolamine salt has the therapeutic effects on insulin deficiency of type 1 diabetes mice and muscle wasting induced by doxorubicin. Therefore, we aim to investigate the therapeutic effects of niclosamide ethanolamine salt on diabetes-induced muscle wasting and to explore whether the mechanism is associated with muscle autophagy.

**Methods:**

Type 1 diabetes mice were induced by intraperitoneal injection of streptozotocin, then were fed with regular diet supplemented with 10 g/kg niclosamide ethanolamine salt. The whole experiment lasted for 8 weeks. At the end of the study, grip strength, weights of tibialis anterior, gastrocnemius, soleus, and extensor digitorum longus muscle were measured. Tibialis anterior muscles stained with PAS were used for evaluating the fiber cross sectional area. Immunofluorescence analysis of myosin heavy chain expression in extensor digitorum longus and soleus muscle was used for determining the composition of the muscle fiber type. Electronic microscopy was applied to observe the autophagy in the atrophied muscle. Serum insulin levels and fasting blood glucose were also measured. Tissues of gastrocnemius muscle were used for detecting the expression of the proteins related to autophagy.

**Results:**

In this study, we found that niclosamide ethanolamine salt could ameliorate muscle atrophy in the type 1 diabetes mice as well, such as enhancing the declined grip strength, improving limb weight and increasing the numbers of glycolytic muscle fiber. Electron microscopy also confirmed that there did exist abundant autophagic vacuoles in the atrophied muscle of the type 1 diabetes mice. Specifically, niclosamide ethanolamine salt could reduce the over expression of autophagy-related proteins, including p-AMPK (Thr172), FoxO3a, p-ULK1 (Ser555), LC3B II, and p-p38 in gastrocnemius muscle of the type 1 diabetes mice.

**Conclusion:**

Niclosamide ethanolamine salt could ameliorate muscle wasting. The mechanisms underlying might be associated with inhibition of muscle autophagy.

## Background

Global diabetes prevalence has been increased rapidly in recent decades. It was estimated that there were 463 million people suffering from diabetes in 2019, and the number is assumed to reach as many as 700 million in 2045 [1]. In the past years, researches on the complications of diabetes have mostly focused on vascular diseases [2, 3]. Accumulating evidences indicated that accelerated loss of muscle mass and strength is also a devastating complication of diabetes [4],which might lead to slow movements, unstable gait, and even frequent falls. What is more, alterations of the biomechanics of the feet caused by muscle atrophy might increase the risk of developing a foot ulcer in diabetes [5, 6]. Therefore, studies investigating the pathogenesis and exploring new medications for diabetes-related muscle atrophy are in an urgent need.

In diabetes, muscle atrophy might take place due to inflammation, hyperglycemia, insulin deficiency, autophagy activation, and ubiquitin-proteasome degradation. However, protein degradation with a net loss of muscle mass is the crucial feature in atrophic muscle. The autophagy-lysosome systems are one of the major protein degradation pathways and proved to contribute to muscle atrophy [7]. Insulin, which is key in the process of glucose uptake, plays a crucial role in protein synthesis and degradation in muscle [8]. Accumulating evidences showed that the glucose starvation by insulin deficiency might trigger the muscle autophagy.

Our previous study found that niclosamide ethanolamine salt (NEN), a classic anthelmintic drug approved by FDA, can improve the declined insulin level and body weight of streptozotocin (STZ)-induced diabetic mice [9]. More important, NEN can improve the muscle wasting induced by doxorubicin [10]. However, the effects of NEN on diabetes-related muscle atrophy are not yet clear. Therefore, this study aims to investigate the therapeutic effects of NEN on diabetes-induced muscle atrophy and to explore whether the mechanism is associated with muscle autophagy.

## Methods

### Animal model

Animal studies were approved by the Guangzhou University of Chinese Medicine Institutional Animal Care and Use Committee and were performed under protocols in accordance with relevant guidelines and regulations. Male C57BL/6J mice were purchased from Guangdong Medical Laboratory Animal Center and were housed in the Laboratory Animal Center of Shenzhen Graduate School of Peking University. The type 1 diabetes (T1D) mice were induced by the administration of multiple low doses of STZ (Sigma-Aldrich, St. Louis, MO, USA) dissolved in citrate buffer via intra-peritoneal injection (55 mg/kg body weight per day) for 5 consecutive days. Normal control (T1D-ctrl) mice were intra-peritoneally injected with an equal volume of citrate buffer. The T1D mice were randomly allocated into T1D group and T1D + NEN group according to the fasting blood glucose at the 9th day after the last injection of STZ. Mice in T1D-ctrl and T1D groups were fed with regular diet as before, while the T1D + NEN group were fed with a regular diet supplemented with 10 g/kg NEN. The whole treatment lasted for 8 weeks.

### Grip strength test

Mice’s limb grip strength was measured by using a dynamometer for mice (ZH-YLS-13A, Anhui Zhenghua Biological Instrument Equipment Co. Ltd., Huaibei, China) the day before being sacrificed. Limb grip strength was performed according to the manufacturer’s instructions. The PC interface software automatically sensed compression or tension and recorded the peak value (in mV). Calibrate factor was measured by using standard weight (1.98 N). Limb strength (in Newton) was calculated by peak value (in mV) × calibrate factor. For this assay, three measurements were performed for each mouse and average of the results was used for analyzing.

### Blood glucose and body weight measurements

Every 2 weeks, each mouse was weighed and blood samples of them were obtained by tail vein puncture for blood glucose measurements by using a blood glucose meter (Roche, Basel, Switzerland).

### Tissue preparation

At the end of the experiment, mice were sacrificed, then the tibialis anterior (TA), soleus (SOL), extensor digitorum longus (EDL), and gastrocnemius (GAs) were dissected and blotted on paper, and then weighed immediately. TA muscle tissues were fixed in 10% formalin for fiber cross-sectional area determination. EDL muscle tissues (sized 1 mm^3^) were fixed in 2.5% glutaraldehyde and then were post-fixed in 1% osmic acid for transmission electronic microscopy (TEM) examination. The GAs muscle tissues were immediately snap-frozen in liquid nitrogen and stored at − 80 °C for later analysis. The SOL muscles and EDL muscles were firstly embedded in O.C.T. compound (Tissue-Tek, Sakura Finetek, USA), then frozen in liquid nitrogen-cooled isopentane, and lastly stored at – 80 °C for fiber type determination.

### Fiber cross-sectional area and fiber size distribution determination

The muscles were photographed by digital camera. Paraffin-embedded TA muscle sections were stained with periodic acid-Schiff (PAS). At least 40% of all fibers within a muscle cross section (about 550–1500 fibers) were outlined to evaluate muscle fiber cross-sectional area and the fiber size distribution by using ImageJ Software (National Institutes of Health, Bethesda, MD, USA).

### Fiber type determination

The prepared SOL and EDL tissues were cut into 10-μm-thick cryo-sections with a cryostat (CM1950, Leica, Germany) maintained at − 20 °C, then immunofluorescence analysis of MHC expression was performed in the procedures as described previously [10]. Primary antibodies against MHC-I (BA-F8), MHC-IIa (SC-71) and MHC-IIb (BF-F3) were purchased from the Developmental Studies Hybridoma Bank (University of Iowa, National Institutes of Health, USA), whereas secondary antibodies were purchased from Invitrogen (USA). The resulting images were visualized and were captured on a confocal microscopy (LSM710, Carl Zeiss, Oberkochen, Germany). Individual images were taken across the entire cross-section, then were assembled into a composite panoramic image with Photoshop 7.0 (Adobe, USA). All fibers within the entire image were characterized for fiber type analysis.

### Electronic microscopy

TEM images were photographed by JEM-1400(JEOL, Tokyo, Japan). Autophagic vacuoles in inter-myofibrillar area and sub-sarcolemmal area were observed and photographed.

### Enzyme-linked immunosorbent assay (ELISA)

Enzyme-linked immunosorbent assay kits were used to measure serum insulin (Merck Millipore, Danvers, MA, USA) according to the manufacturer’s instructions.

### Immunoblotting analysis

Snap-frozen GAs muscle tissues were homogenized in lysis buffer and prepared in sample loading buffer (Bio-Rad, Hercules, CA, USA). Lysate proteins were separated on a 10% SDS-PAGE gels and then transferred to polyvinylidenedifluoride (PVDF) membranes (Merck Millipore, Danvers, MA, USA). After blocking in TBS buffer containing 5% non-fat dry milk for 1 h at room temperature, the membranes were incubated and gently shaken overnight at 4 °C with primary antibodies. After washing with TBS, the membranes were incubated with secondary antibodies for 1 h at room temperature with shaking. After washing, the protein bands were detected and analyzed by a ChemiDoc™ MP Imaging System (Bio-Rad, Hercules, CA, USA). Glyceraldehyde-3-phosphate dehydrogenase (GAPDH) was used as the loading control. The results are expressed as the integrated optical density relative to GAPDH. Primary antibodies against GAPDH were used as the loading control. The results are expressed as the integrated optical density relative to GAPDH. Primary antibodies against p-ULK1(ser555), ULK1, p-AMPK (Thr172), AMPK, LC3B, p-p38 MAPK(Thr180/Tyr182), p38 MAPK, and FoxO3a were purchased from Cell Signaling Technology (Danvers, MA, USA). Primary antibody against GAPDH was from Proteintech Group, Inc. (Chicago, IL, USA).

### Statistical analysis

Data were expressed as mean ± SD. Statistical differences between two groups were analyzed using unpaired Student’s *t* tests. Repeated measures analyses of variance (ANOVA) were conducted for the blood glucose and body weight data, the effects being group (T1D-ctrl vs. T1D, T1D vs T1D + NEN) and week (week 0, 2, 4, 6, 8, 10). Post hoc testing was performed using *Bonferroni*. Statistical analysis was performed using SPSS statistical software, version 16.0, and *P* < 0.05 was considered statistically significant.

## Results

### NEN prevented muscle weakness in the T1D mice

**Fig. 1:**
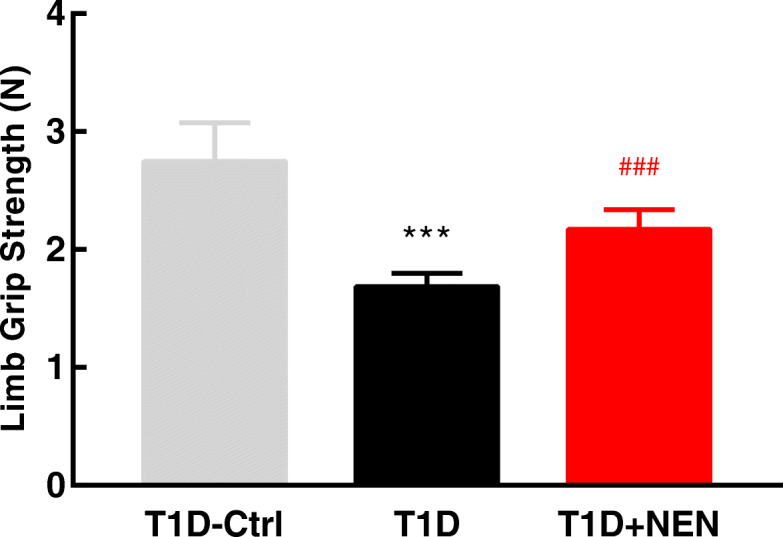
NEN improved the limb grip strength of the T1D mice. Bar graph of limb grip strength. *n* = 6 per group. ^***^P < 0.001 vs. the T1D-Ctrl group. ^###^*P* < 0.001 vs. the T1D group

In order to detect whether the muscle function of the T1D mice is affected by NEN, mice were subjected to grip strength assessment. It turned out that the T1D mice exhibited declined grip strength, while NEN treatment could enhance the grip strength of the T1D mice (Fig. 1).

### NEN restored body weight and improved limb muscle atrophy in the T1D mice

**Fig. 2:**
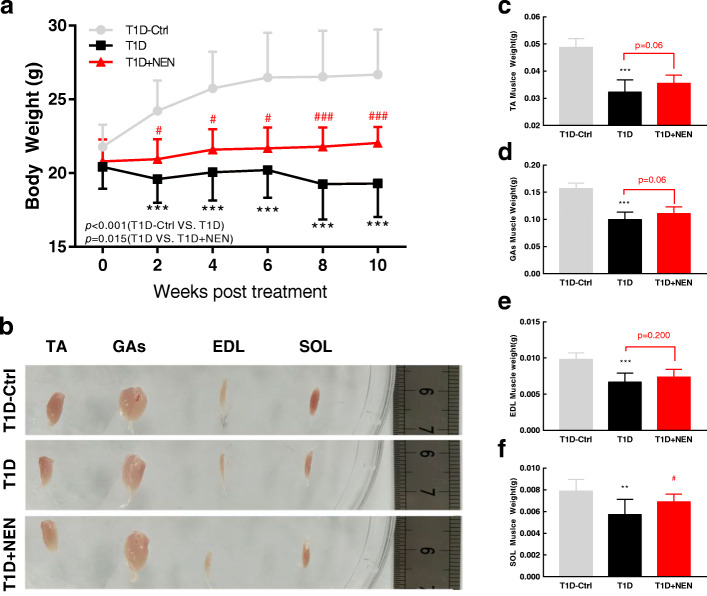
NEN restored body weight and muscle mass in the T1D mice. **a** Body weight of mice was measured every 2 weeks; Repeated measures analyses of variance (ANOVA) were conducted, then post hoc testing was performed using Bonferroni; the T1D mice exhibited decreased bodyweight significantly compared with normal control mice from the 9th day after the injection of STZ (*P* = 0.000 < 0.001); following the treatment with NEN, body weight of theT1D mice were increasing gradually (*P* = 0.015 < 0.05). **b** Representative images of muscle. **c** Weight of TA muscle. **d** Weight of GAs muscle. **e** Weight of EDL muscle. **f** Weight of SOL muscle. ^**^P < 0.01 and ^***^P < 0.001 vs. the T1D-Ctrl group. ^#^*P* < 0.05 and ^###^*P* < 0.001 vs. the T1D group. *n* = 6 in each group

In addition to enhancing muscle strength, NEN also had therapeutic effect on skeletal muscle atrophy in theT1D mice. The T1D mice exhibited decreased bodyweight significantly compared with normal control mice from the 9th day after the injection of STZ (*P* = 0.000 < 0.001). Following the treatment with NEN, body weight of the T1D mice were increasing gradually (*P* = 0.015<0.05) (Fig. 2a). At the end of experiment, hindlimb muscles of the T1D mice were smaller than those of the control mice, but were improved by the treatment with NEN (Fig. 2b). The analysis of individual low hindlimb muscles revealed that the weights of TA, GAs, EDL, and SOL were decreased in theT1D mice group compared with control group significantly, while NEN could increase the muscle mass of TA, GAs and SOL (Fig. 2c–f).

### NEN increased theT1D mice’s muscle fiber size

**Fig. 3:**
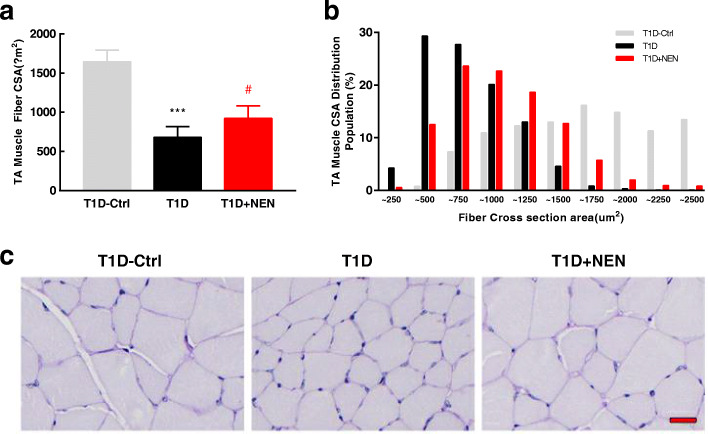
NEN improved the muscle fiber cross-sectional area. **a** TA muscle fiber cross-sectional area in each group. **b** Frequency histograms showing the distribution of cross-sectional area of TA muscle fibers. **c** Representative images of fiber size alteration (PAS stained. scale bar, 20 μm). ^***^P < 0.001 vs. the T1D-Ctrl group. ^#^*P* < 0.05 vs. the T1D group. *n* = 6 in each group

We further measured cross-sectional area of skeletal muscle fibers in the mice. Likewise, the mean cross-sectional area of TA muscle in the T1D mice had shrunken remarkably, and NEN did increase the mean cross-sectional area of TA muscle fiber (Fig. 3a, c). In addition, the cross-sectional area distribution of TA muscle in the T1D mice developed a shift towards smaller fibers, and NEN could normalized this change as well (Fig. 3b).

### NEN restored glycolytic muscle fiber in the T1D mice

**Fig. 4:**
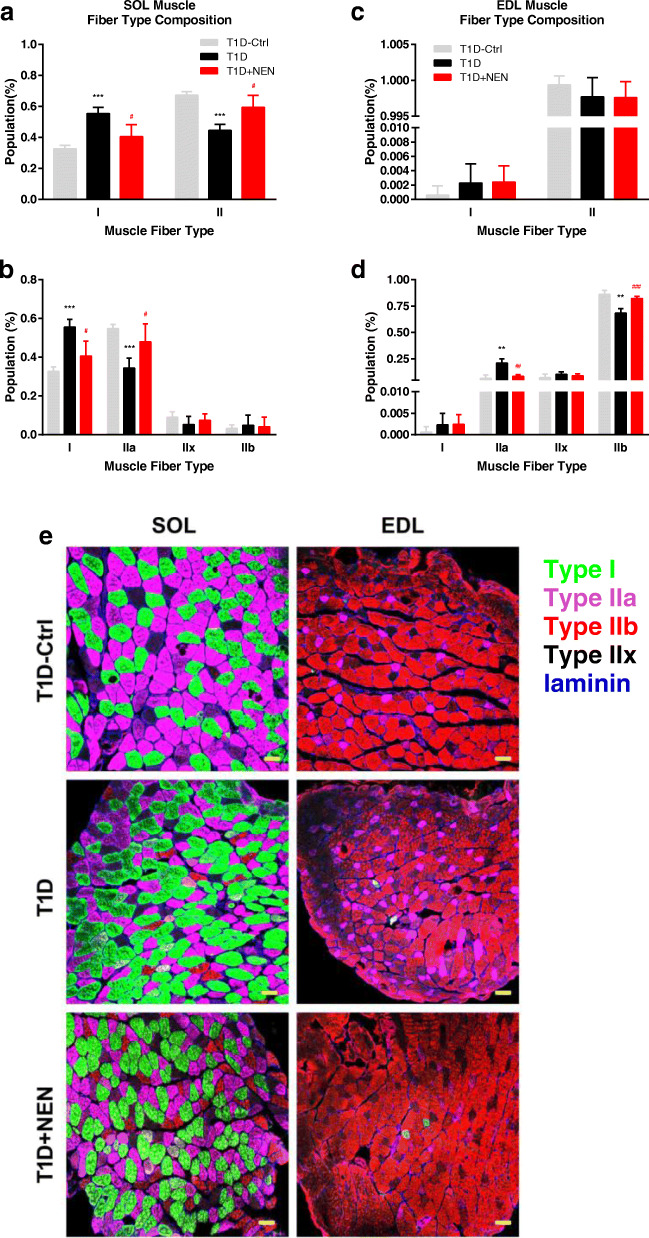
Muscle fiber type composition of SOL and EDL with immunofluorescence analysis. **a**, **b** SOL muscle fiber type composition. **c**, **d** EDL muscle fiber type composition. **e** Representative images of muscle fiber type composition (scale bar, 50 μm). *n* = 6 per group. ^**^P < 0.01, and ^***^P < 0.001 vs. the T1D-Ctrl group. ^#^*P* < 0.05, ^##^*P* < 0.01 and ^###^*P* < 0.001 vs. the T1D group

As shown in Fig. 4a, c, e, the numbers of type II fiber were decreased in SOL muscle obviously but no change in EDL muscle in theT1D mice, and NEN could increase the numbers of type II fiber in SOL muscle. Furthermore, the composition of type II glycolytic fibers was analyzed, and it showed that the fiber subtypes were altered in the T1D mice. Type IIa fibers decreased in the SOL muscle (Fig. 4b, e), while type IIb fibers were less in the EDL muscle (Fig. 4d, e). Interestingly, NEN could restore these fiber subtypes (Fig. 4b, d, e).

### NEN improved insulin deficiency and energy shortage in T1D mice

**Fig. 5:**
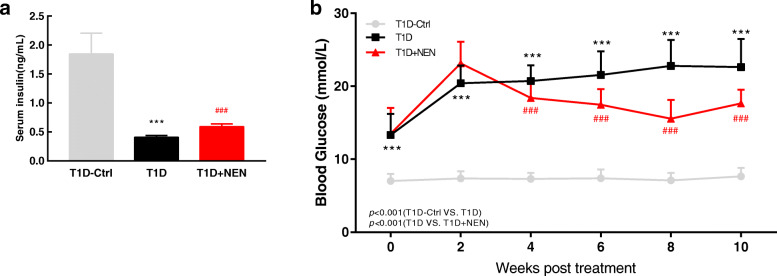
Serum insulin and blood glucose levels of mice from each group. **a** Serum insulin levels at 12 weeks post-treatment with NEN. **b** Fasting blood glucose was measured every 2 weeks in the whole experiment; repeated measures analyses of variance (ANOVA) were conducted, then post hoc testing was performed using Bonferroni; the T1D mice exhibited increased blood glucose significantly compared with normal control mice through the whole experiment (*P* = 0.000 < 0.001); following the treatment with NEN, blood glucose of the T1D mice started to decrease at the 4th week (*P* = 0.000 < 0.001). ^***^P < 0.001 vs. the T1D-Ctrl group. ^###^*P* < 0.001 vs. the T1D group. *n* = 6 in each group

Our previous study suggested that the protective effects of NEN on the diabetic mice might be due to improving β-cell function or inducing the α-to-β-like cell conversion [11, 12]. Consistent with previous studies, the serum insulin level of the T1D mice was significantly lower than that of the control group and following NEN treatment could raise it (Fig. 5a). In consequence, T1D + NEN group showed lower blood glucose than the T1D group (*P* = 0.000 < 0.001) (Fig. 5b). The expression of p-AMPK (Thr172) increased remarkably as a consequence of insulin deficiency in T1D mice’s muscle, which indicated that there was short of energy supply. NEN could decrease the expression of p-AMPK (Thr172) (Fig. 7a, b, c).

### NEN suppressed glucose starvation-induced muscle autophagy in T1D mice

**Fig. 6:**
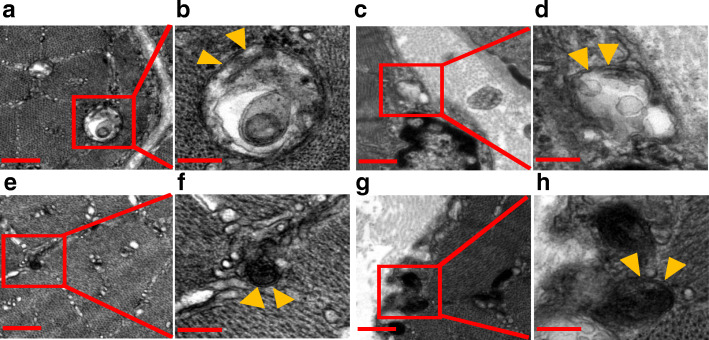
Ultra-structure of autophagic vacuoles in EDL muscle in T1D mice. **a**, **b** Early autophagic vacuole in inter-myofibrillar area. **c**, **d** Early autophagic vacuole in sub-sarcolemmal area. **e**, **f** Late autophagic vacuole in inter-myofibrillar area. **g**, **h** Late autophagic vacuoles in sub-sarcolemmal area. The arrowheads indicate the two limiting membranes in autophagic vacuoles. Scale bar 500 nm for **a**, **c**, **e**, **g**, 200 nm for **b**, **d, f**, **h**

**Fig. 7:**
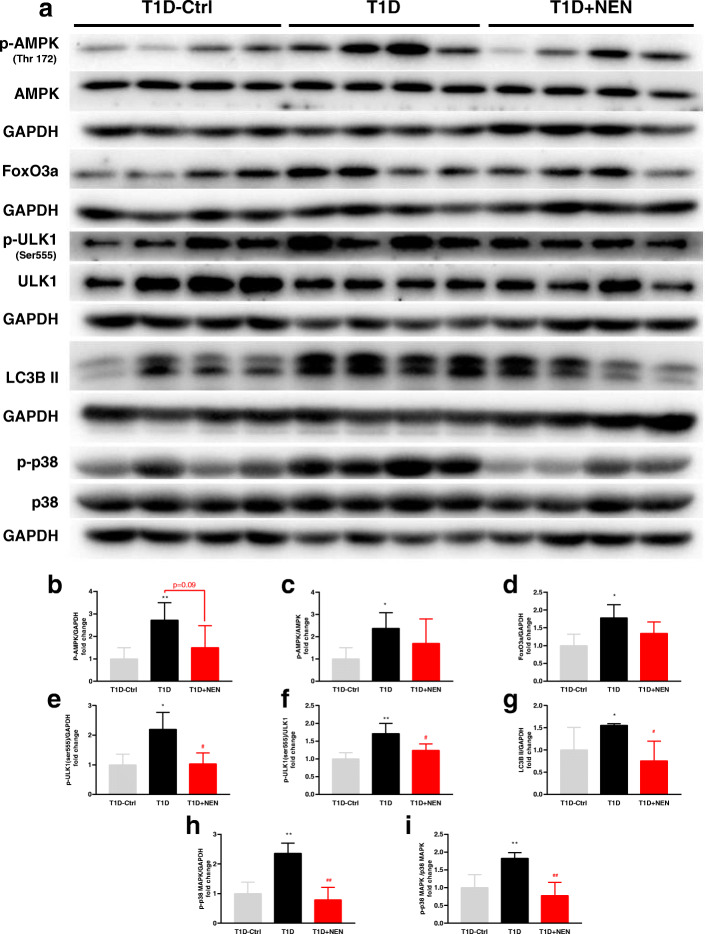
Autophagy-related proteins expression in GAs muscle tissues. **a** Western blot images of autophagy-related proteins in GAs muscle tissues of each group. Bar graphs showing the fold change of p-AMPK/GAPDH (**b**), p-AMPK/AMPK (**c**), FoxO3a/GAPDH (**d**), p-ULK1(ser555)/GAPDH (**e**), p-ULK1(ser555)/ULK1(f), LC3B II/GAPDH (**g**), p-p38/GAPDH (**h**), and p-p38/p38 MAPK (**i**) expression in GAs muscle tissues. ^*^P < 0.05 and ^**^P < 0.01 vs. the T1D-Ctrl group. ^#^*P* < 0.05 and ^##^*P* < 0.01 vs. the T1D group. *n* = 4 in each group

Electron microscopy revealed that autophagic vacuoles were abundant within inter-myofibrillar and sub-sarcolemmal area (Fig. 6a–h). Accordingly, we next investigated the expression of autophagy-related proteins to see if there was excessive autophagy in the muscle of the T1D mice. It turned out that the protein expressions of FoxO3a, p-ULK1 (Ser555), LC3B-II, and p-p38 MAPK (Thr180/Tyr182) were greatly elevated in the T1D mice. Moreover, the excessive expressions of the above proteins in the T1D mice were diminished in T1D + NEN group. These indicated that NEN could ameliorate muscle autophagy (Fig. 7a–i).

## Discussion

This study indicates that NEN prevents muscle wasting in the T1D mice and the mechanism underlying might be associated with the inhibition of muscle autophagy induced by the glucose starvation.

It is reported that weight gains have benefit effects on the enhancement of muscle strength [13]. Consistent with our previous study [10], this study showed that NEN could counteract the decrease of body weight, muscle mass along with enhancing hindlimb grip strength. It suggested that the effects of NEN on muscle wasting might benefit from increasing the weight of T1D mice muscle.

Skeletal muscle fibers are characterized as one type of slow-twitch fiber (type I) and three types of fast-twitch fibers (type IIa, type IIx/d, and type IIb), of which type IIb fibers are primarily glycolytic. In diabetes, fibers might change from fast-twitch type to slow-twitch type with preferential atrophy of type II fiber [14, 15], as type II fiber were more vulnerable to nutritional deficiencies [16]. Loss of glycolytic muscles might lead to grip strength declined [17]. In this study, the fibers of TA muscle, which was almost composed of type II fibers, were atrophy obviously in the T1D mice. We also found that the fibers of fast/glycolytic were decreased in SOL muscle and EDL muscle. Taken together, we implied that the protection of NEN on diabetes-related muscle wasting might be partly due to the restoration of type II fiber.

Skeletal muscle is the prominent organ for insulin-mediated glucose uptake [18]. Glucose starvation in skeletal muscle caused by insulin deficiency could result in significant reduction in muscle mitochondrial ATP production rate [19]. Interestingly, autophagy can activate bulk protein degradation to harvest amino acids as a fuel for ATP production through the tricarboxylic acid (TCA) cycle to maintain the energy balance [20]. Therefore, in insulin deficiency T1D mice, autophagy plays a crucial role in muscle atrophy, which might be activated by the energy shortage to use amino acid as a substitute for glucose. AMPK is a sensor of intracellular energy, which can be activated by any mechanisms that disrupt ATP generation [21]. Studies showed that AMPK activation can promote skeletal muscle cells autophagy by activating FoxO3a and ULK1 [22, 23]. Accumulating evidences indicate that FoxO3a is the main transcriptional regulator of autophagy by controlling a broad range of atrophy-related genes, including Fbxo32 (Atrogin-1) and Trim63 (MuRF-1), and other autophagy genes [7, 11]. ULK1 is one of the essential inductors of the autophagy pathway, which initiates the formation of the autophagosome [24]. Our study showed that T1D mice treated with NEN did develop less expression of p-AMPK (Thr172), FoxO3a, and p-ULK1 (Ser555) than T1D mice.

P38 MAPK is also known to regulate autophagy in skeletal muscle [25, 26] due to the over phosphorylation in skeletal muscle under variety of cellular stresses, including endurance exercise and fasted state [27]. Our previous study also found that NEN can prevent muscle atrophy by inhibition of p38 MAPK/FoxO3a activation in mice exposed to doxorubicin [10]. Similarly, as the above results showed, NEN treatment also inhibited the over-activation of p38MAPK in T1D mice. The level of LC3BII, a marker of autophagosome presence [28], was reduced by NEN treatment in T1D mice in this study. The result further prompted that autophagy was suppressed by NEN.

## Conclusion

In summary, we concluded that NEN could ameliorate muscle wasting. The mechanisms underlying might be associated with inhibition of muscle autophagy.

## Data Availability

The datasets used and/or analyzed during the current study are available from the corresponding author on reasonable request.

## Abbreviations

NEN: Niclosamide ethanolamine salt
STZ: Streptozotocin
T1D: Type 1 diabetes
SOL: Soleus
TA: Tibialis anterior
EDL: Extensor distal longus
GAs: Gastrocnemius
GAPDH: Glyceraldehyde-3-phosphate dehydrogenase

## References

[1] Harding JL, Pavkov ME, Magliano DJ, Shaw JE, Gregg EW (2019). Global trends in diabetes complications: a review of current evidence. Diabetologia..

[2] Williams R, Karuranga S, Malanda B, Saeedi P, Basit A, Besançon S (2020). Global and regional estimates and projections of diabetes-related health expenditure: results from the International Diabetes Federation Diabetes Atlas, 9th edition. Diabetes Res Clin Pract.

[3] Gregg EW, Sattar N, Ali MK (2016). The changing face of diabetes complications. Lancet Diabetes Endocrinol.

[4] Klionsky DJ, Abeliovich H, Agostinis P, Agrawal DK, Aliev G, Askew DS, Baba M, Baehrecke EH, Bahr BA, Ballabio A, Bamber BA, Bassham DC, Bergamini E, Bi X, Biard-Piechaczyk M, Blum JS, Bredesen DE, Brodsky JL, Brumell JH, Brunk UT, Bursch W, Camougrand N, Cebollero E, Cecconi F, Chen Y, Chin LS, Choi A, Chu CT, Chung J, Clarke PG, Clark RS, Clarke SG, Clavé C, Cleveland JL, Codogno P, Colombo MI, Coto-Montes A, Cregg JM, Cuervo AM, Debnath J, Demarchi F, Dennis PB, Dennis PA, Deretic V, Devenish RJ, di Sano F, Dice JF, Difiglia M, Dinesh-Kumar S, Distelhorst CW, Djavaheri-Mergny M, Dorsey FC, Dröge W, Dron M, Dunn WA Jr, Duszenko M, Eissa NT, Elazar Z, Esclatine A, Eskelinen EL, Fésüs L, Finley KD, Fuentes JM, Fueyo J, Fujisaki K, Galliot B, Gao FB, Gewirtz DA, Gibson SB, Gohla A, Goldberg AL, Gonzalez R, González-Estévez C, Gorski S, Gottlieb RA, Häussinger D, He YW, Heidenreich K, Hill JA, Høyer-Hansen M, Hu X, Huang WP, Iwasaki A, Jäättelä M, Jackson WT, Jiang X, Jin S, Johansen T, Jung JU, Kadowaki M, Kang C, Kelekar A, Kessel DH, Kiel JA, Kim HP, Kimchi A, Kinsella TJ, Kiselyov K, Kitamoto K, Knecht E, Komatsu M, Kominami E, Kondo S, Kovács AL, Kroemer G, Kuan CY, Kumar R, Kundu M, Landry J, Laporte M, le W, Lei HY, Lenardo MJ, Levine B, Lieberman A, Lim KL, Lin FC, Liou W, Liu LF, Lopez-Berestein G, López-Otín C, Lu B, Macleod KF, Malorni W, Martinet W, Matsuoka K, Mautner J, Meijer AJ, Meléndez A, Michels P, Miotto G, Mistiaen WP, Mizushima N, Mograbi B, Monastyrska I, Moore MN, Moreira PI, Moriyasu Y, Motyl T, Münz C, Murphy LO, Naqvi NI, Neufeld TP, Nishino I, Nixon RA, Noda T, Nürnberg B, Ogawa M, Oleinick NL, Olsen LJ, Ozpolat B, Paglin S, Palmer GE, Papassideri I, Parkes M, Perlmutter DH, Perry G, Piacentini M, Pinkas-Kramarski R, Prescott M, Proikas-Cezanne T, Raben N, Rami A, Reggiori F, Rohrer B, Rubinsztein DC, Ryan KM, Sadoshima J, Sakagami H, Sakai Y, Sandri M, Sasakawa C, Sass M, Schneider C, Seglen PO, Seleverstov O, Settleman J, Shacka JJ, Shapiro IM, Sibirny A, Silva-Zacarin EC, Simon HU, Simone C, Simonsen A, Smith MA, Spanel-Borowski K, Srinivas V, Steeves M, Stenmark H, Stromhaug PE, Subauste CS, Sugimoto S, Sulzer D, Suzuki T, Swanson MS, Tabas I, Takeshita F, Talbot NJ, Tallóczy Z, Tanaka K, Tanaka K, Tanida I, Taylor GS, Taylor JP, Terman A, Tettamanti G, Thompson CB, Thumm M, Tolkovsky AM, Tooze SA, Truant R, Tumanovska LV, Uchiyama Y, Ueno T, Uzcátegui NL, van der Klei I, Vaquero EC, Vellai T, Vogel MW, Wang HG, Webster P, Wiley JW, Xi Z, Xiao G, Yahalom J, Yang JM, Yap G, Yin XM, Yoshimori T, Yu L, Yue Z, Yuzaki M, Zabirnyk O, Zheng X, Zhu X, Deter RL (2008). Guidelines for the use and interpretation of assays for monitoring autophagy in higher eukaryotes. Autophagy..

[5] Kim J, Won KJ, Lee HM, Hwang BY, Bae YM, Choi WS, Song H, Lim KW, Lee CK, Kim B (2009). p38 MAPK Participates in muscle-specific RING finger 1-mediated atrophy in cast-immobilized rat gastrocnemius muscle. Korean J Physiol Pharmacol.

[6] Doyle A, Zhang G, Abdel Fattah EA, Eissa NT, Li YP (2011). Toll-like receptor 4 mediates lipopolysaccharide-induced muscle catabolism via coordinate activation of ubiquitin-proteasome and autophagy-lysosome pathways. FASEB J.

[7] McClung JM, Judge AR, Powers SK, Yan Z (2010). p38 MAPK links oxidative stress to autophagy-related gene expression in cachectic muscle wasting. Am J Physiol Cell Physiol.

[8] Mizushima N, Yoshimori T, Ohsumi Y (2011). The role of Atg proteins in autophagosome formation. Annu Rev Cell Dev Biol.

[9] O'Neill BT, Bhardwaj G, Penniman CM, Krumpoch MT, Suarez Beltran PA, Klaus K (2019). FoxO transcription factors are critical regulators of diabetes-related muscle atrophy. Diabetes..

[10] Zhan H, Wang M, Han P, Yu X, Wang Y, Weng W (2020). Niclosamide ethanolamine prevents muscle wasting by inhibiting p38 MAPK-FoxO3a activation in mice exposed to doxorubicin. Int J Clin Exp Med.

[11] Han P, Shao M, Guo L, Wang W, Song G, Yu X, Zhang C, Ge N, Yi T, Li S, du H, Sun H (2018). Niclosamide ethanolamine improves diabetes and diabetic kidney disease in mice. Am J Transl Res.

[12] Han P, Zhan H, Shao M, Wang W, Song G, Yu X, Zhang C, Ge N, Yi T, Li S, Sun H (2018). Niclosamide ethanolamine improves kidney injury in db/db mice. Diabetes Res Clin Pract.

[13] Kjøbsted R, Hingst JR, Fentz J, Foretz M, Sanz MN, Pehmøller C, Shum M, Marette A, Mounier R, Treebak JT, Wojtaszewski JFP, Viollet B, Lantier L (2018). AMPK in skeletal muscle function and metabolism. FASEB J.

[14] Armstrong RB, Gollnick PD, Ianuzzo CD (1975). Histochemical properties of skeletal muscle fibers in streptozotocin-diabetic rats. Cell Tissue Res.

[15] Schiaffino S, Dyar KA, Ciciliot S, Blaauw B, Sandri M (2013). Mechanisms regulating skeletal muscle growth and atrophy. FEBS J.

[16] Egan DF, Shackelford DB, Mihaylova MM, Gelino S, Kohnz RA, Mair W (2011). Phosphorylation of ULK1 (hATG1) by AMP-activated protein kinase connects energy sensing to mitophagy. Science (New York, NY).

[17] Sanchez AM, Csibi A, Raibon A, Cornille K, Gay S, Bernardi H (2012). AMPK promotes skeletal muscle autophagy through activation of forkhead FoxO3a and interaction with Ulk1. J Cell Biochem.

[18] Kim KH, Lee MS (2014). Autophagy as a crosstalk mediator of metabolic organs in regulation of energy metabolism. Rev Endocr Metab Disord.

[19] Karakelides H, Asmann YW, Bigelow ML, Short KR, Dhatariya K, Coenen-Schimke J, Kahl J, Mukhopadhyay D, Nair KS (2007). Effect of insulin deprivation on muscle mitochondrial ATP production and gene transcript levels in type 1 diabetic subjects. Diabetes..

[20] Deshmukh AS (2016). Insulin-stimulated glucose uptake in healthy and insulin-resistant skeletal muscle. Horm Mol Biol Clin Invest.

[21] Zibellini J, Seimon RV, Lee CM, Gibson AA, Hsu MS, Sainsbury A (2016). Effect of diet-induced weight loss on muscle strength in adults with overweight or obesity - a systematic review and meta-analysis of clinical trials. Obes Rev.

[22] Choi S, Jeong HJ, Kim H, Choi D, Cho SC, Seong JK, Koo SH, Kang JS (2019). Skeletal muscle-specific Prmt1 deletion causes muscle atrophy via deregulation of the PRMT6-FOXO3 axis. Autophagy..

[23] Wang Y, Pessin JE (2013). Mechanisms for fiber-type specificity of skeletal muscle atrophy. Curr Opin Clin Nutr Metab Care.

[24] Workeneh B, Bajaj M (2013). The regulation of muscle protein turnover in diabetes. Int J Biochem Cell Biol.

[25] Mammucari C, Milan G, Romanello V, Masiero E, Rudolf R, Del Piccolo P (2007). FoxO3 controls autophagy in skeletal muscle in vivo. Cell Metab.

[26] Andersen H (2012). Motor dysfunction in diabetes. Diabetes Metab Res Rev.

[27] Gorial FI, Sayyid OS, Al Obaidi SA (2020). Prevalence of sarcopenia in sample of Iraqi patients with type 2 diabetes mellitus: A hospital based study. Diabetes Metab Syndr.

[28] Kalyani RR, Corriere M, Ferrucci L (2014). Age-related and disease-related muscle loss: the effect of diabetes, obesity, and other diseases. Lancet Diabetes Endocrinol.

